# Factors associated with disability in patients with rheumatoid arthritis with persistent moderate disease activity: a retrospective cohort study

**DOI:** 10.1186/s41927-020-00161-4

**Published:** 2020-10-21

**Authors:** Ian C. Scott, Julie Mount, Jane Barry, Bruce Kirkham

**Affiliations:** 1 Primary Care Centre Versus Arthritis, School of Primary, Community and Social Care, Keele University, Keele, Staffordshire ST5 5BG UK; 2 Haywood Academic Rheumatology Centre, Haywood Hospital, Midlands Partnership NHS Foundation Trust, High Lane, Burslem, Staffordshire ST6 7AG UK; 3 Eli Lilly and Company, Priestly Road, Basingstoke, Hampshire, RG24 9NL UK; 4 Department of Rheumatology, Guy’s and St. Thomas’ NHS Foundation Trust, Great Maze Pond, London, SE1 9RT UK

**Keywords:** Rheumatoid arthritis, Moderate disease activity, Disability, Quality of life, Pain

## Abstract

**Background:**

Many patients with rheumatoid arthritis (RA) do not attain remission/low disease activity, remaining in a moderate disease activity state (MDAS) with ongoing disability and impaired quality of life (QoL). If patients in persistent MDAS with poor future outcomes could be prospectively identified, they could arguably be treated more intensively. We evaluated baseline factors predicting function (Health Assessment Questionnaire-Disability Index [HAQ-DI] scores) and QoL (3-level EuroQol-5 dimensions questionnaire [EQ-5D-3L] index scores) at 12 months in patients with RA in persistent MDAS in a real-world setting.

**Methods:**

Patients with persistent MDAS (Disease Activity Score for 28-joint count based on erythrocyte sedimentation rate [DAS28-ESR] 3.2–5.1 on at least two consecutive outpatient appointments over 12 months) were identified retrospectively from Guy’s Hospital RA Centre and analysed in two groups: (1) biologic naïve at baseline or (2) receiving/ever received biologics. The baseline timepoint was the second-visit MDAS DAS28-ESR score; the endpoint was the closest visit to 12 months. Linear regression analyses evaluated relationships between baseline variables and (1) 12-month HAQ-DI scores, (2) 12-month rank-transformed EQ-5D-3L index scores, (3) 12-month changes in HAQ-DI scores, and (4) 12-month changes in EQ-5D-3L index scores.

**Results:**

The analysis included 207 biologic-naïve and 188 biologic-experienced patients. All patients had moderate disability (mean HAQ-DI 1.21 and 1.46) and impaired QoL (mean EQ-5D-3L index scores 0.52 and 0.50). Many reported moderate/severe pain (93 and 96%) and showed little change in HAQ-DI and EQ-5D-3L index scores over 12 months. In both biologic-naïve and biologic-experienced groups, multivariate analysis revealed a significant association between baseline HAQ-DI scores and endpoint HAQ-DI scores (β = 0.67, *P* < 0.001 and β = 0.76, *P* < 0.001, respectively), 12-month changes in HAQ-DI scores (both β = − 0.21, *P* < 0.001), and 12-month EQ-5D-3L index scores (β = − 0.57, *P* < 0.001 and β = − 0.29, *P* = 0.004, respectively). Baseline EQ-5D-3L index scores were significantly associated with 12-month changes in EQ-5D-3L index scores in both groups (β = − 0.73, *P* < 0.001 and β = − 0.40, *P* = 0.003, respectively).

**Conclusions:**

Patients with RA in persistent MDAS experience substantial ongoing physical disability, poor QoL, and pain. HAQ-DI is an important predictor of future disability and reduced QoL, supporting current national recommendations to measure HAQ-DI in routine care.

## Background

The established approach to managing patients with rheumatoid arthritis (RA) is treat-to-target (T2T) [1]. This focuses on the gradual up-titration of synthetic disease-modifying anti-rheumatic drugs (DMARDs), followed by the introduction of biologics and targeted synthetic DMARDs, until the goals of sustained remission or a low disease activity state (LDAS) are attained [2, 3]. In routine clinical practice, remission and LDAS are often defined as the attainment of a Disease Activity Score for 28-joint count based on erythrocyte sedimentation rate (DAS28-ESR) of < 2.6 and ≥ 2.6 to < 3.2, respectively.

A key challenge in implementing T2T in routine care is that a substantial minority of patients do not reach remission or LDAS and instead remain in a moderate disease activity state (MDAS), defined as a DAS28-ESR of ≥3.2 to ≤5.1. This was exemplified in two separate observational cohort studies of patients with early RA. First, in the UK-based Early RA Study of 2045 patients with DAS28 recorded at least twice between the first and fifth year of follow-up, 47% had mean DAS28 scores over time in the MDAS range [4]. Second, in the North America-based CATCH study, which employed group-based trajectory modelling, 10% of 1586 patients were initially in a high disease activity state (HDAS) and only improved to MDAS over 2 years [5].

There is emerging evidence that patients in persistent MDAS have worse outcomes than those attaining LDAS or remission, exhibiting higher levels of joint damage [4], worse function [4, 6], and lower health-related quality of life (HRQoL) [5]. If it were possible to identify patients in persistent MDAS who are most likely to have poor outcomes, then an argument could be made for treating such individuals more intensively. Such treatment could include biologics or targeted synthetic DMARDs, which in the UK are currently restricted to patients in HDAS (defined as DAS28-ESR > 5.1) [7], and intensive physical therapy [8].

To this end, we undertook a retrospective analysis using records of patients with RA in persistent MDAS managed using a T2T approach in a routine UK national health service setting. Our primary aim was to identify factors predicting patient functional outcomes at 12 months. Secondary aims were to identify factors predicting patient HRQoL at 12 months and to characterise pain, disability, and HRQoL in patients in persistent MDAS.

## Methods

### Subjects

We retrospectively evaluated patient records from Guy’s Hospital RA Centre, which maintains an electronic healthcare record cohort of patients attending routine rheumatology appointments at Guy’s Hospital (South London) [9, 10]. Since inception of the database in 2006, clinician- and patient-reported outcomes, including DAS28-ESR, Health Assessment Questionnaire-Disability Index (HAQ-DI) [11] and EuroQol 5-dimensions 3-level questionnaire index (EQ-5D-3L) scores (UK value set) [12], have been routinely recorded for patients at each clinic visit. All patients are managed in line with T2T recommendations.

No globally accepted definition of persistent MDAS exists, with previous research in this area using varying descriptions [4, 6]. In this analysis, we considered patients to be in persistent MDAS if they had DAS28-ESR scores of 3.2–5.1 on at least two consecutive outpatient appointments over a 12-month period. For the purposes of this study, patients also had to have recorded HAQ-DI scores at both baseline and final timepoints (to allow the primary study aim to be addressed).

Patients in persistent MDAS were analysed in two separate groups: first, those naïve to biologic DMARDs at baseline and, second, those who were receiving or had previously received biologic DMARDs. The rationale for this sub-categorisation of patients was that the next step in the therapeutic pathway could differ between these patient groups.

### Statistical analysis

For this analysis, the baseline timepoint was the visit at which patients met our definition of ‘persistent MDAS’. Thus, the baseline timepoint was the second of two consecutive outpatient appointments over a 12-month period in which each patient had a DAS28-ESR score of 3.2–5.1. The endpoint was the outpatient visit closest to 12 months after the baseline visit.

Linear regression analyses were used to explore relationships between baseline variables and (1) 12-month HAQ-DI scores, (2) 12-month EQ-5D-3L index scores, (3) 12-month changes in HAQ-DI scores, and (4) 12-month changes in EQ-5D-3L index scores. Rank-transformed 12-month EQ-5D-3L index scores were used, as raw 12-month EQ-5D-3L index scores were not normally distributed. This involved the use of an ordered quantile normalisation transformation approach, a rank-based procedure whereby the values of a vector are mapped to their percentile, which is then mapped to the same percentile of the normal distribution [13].

Baseline variables evaluated in the linear regression models comprised age, sex, ethnicity, disease duration, rheumatoid factor (RF) status, RA therapy (monotherapy or combination synthetic DMARD therapy or no treatment for biologic-naïve patients; DMARD monotherapy, biologic monotherapy, or combination synthetic DMARD and biologic therapy for biologic-experienced patients), corticosteroid use, ESR, DAS28-ESR, swollen joint count (SJC), tender joint count (TJC), HAQ-DI score, Patient Global Assessment of Disease Activity (PtGA), EQ-5D-3L index score, EQ-5D-3L pain score (question 4 of the EQ-5D-3L), and EQ-5D-3L anxiety/depression score (question 5 of the EQ-5D-3L). Due to substantial levels of missing data, we did not include anti-cyclic citrullinated peptide or C-reactive protein levels in the linear regression models, opting for RF and ESR to capture information on serology and the acute-phase response instead.

Univariate linear regression models included either (1) 12-month HAQ-DI scores, (2) 12-month rank-transformed EQ-5D-3L index scores, (3) 12-month change in HAQ-DI scores, or (4) 12-month change in EQ-5D-3L index scores as the response variable and each individual baseline variable as the explanatory variable. Baseline variables with a *P*-value of < 0.1 in univariate models were subsequently included in multivariate regression models as explanatory variables.

Correlation coefficients for variables that may have been affected by strong multicollinearity (DAS28 and its components; EQ-5D-3L index scores and EQ-5D-3L pain and depression/anxiety scores) were checked to ensure they did not meet the pre-defined threshold of > 0.7, which would have precluded entry of both variables into the same model.

For all analyses, *P*-values of < 0.05 were considered statistically significant; no multiplicity adjustments were undertaken. Missing data were not imputed. All analyses were performed using R (version 3.5.3). Rank-based transformations were undertaken using the R package ‘bestNormalise’ [13].

## Results

### Baseline characteristics

From 17,002 patient-visits, we identified 422 patients with RA who met our criteria for persistent MDAS. Of these, 395 had HAQ-DI scores at baseline and final timepoints, and were included in the analysis (207 biologic-naïve and 188 biologic-experienced patients).

In both patient groups, baseline HAQ-DI and EQ-5D-3L index scores indicated moderate disability and reduced HRQoL (Table 1). The majority of patients reported moderate or severe pain on the EQ-5D-3L pain scale at baseline (93% of biologic-naïve and 96% of biologic-experienced patients). Compared with biologic-naïve patients, biologic-experienced patients were younger, showed longer RA duration, were more likely to be RF positive, and had higher rates of corticosteroid use. They also had higher DAS28-ESR and HAQ-DI scores and were more likely to report moderate or severe anxiety/depression at study baseline.

**Table 1:** Baseline characteristics of patients with RA with persistent moderate disease activity

Baseline variable		Biologic naïve (*N* = 207)	Biologic experienced (*N* = 188)
Age (years)		58.5 ± 15.6	55.4 ± 14.1
Female		167 (81)	153 (81)
Ethnicity	White	140 (70)	112 (74)
	Black	33 (17)	25 (17)
	Asian	12 (6)	6 (4)
	Mixed	4 (2)	3 (2)
	Other	9 (5)	5 (3)
Duration of RA (years)		7.0 ± 9.0	21.1 ± 12.0
RF positive		105 (64)	131 (80)
Anti-CCP positive		69 (54)	65 (59)
Corticosteroids		22 (11)	38 (20)
Treatment: Biologic naïve	Monotherapy	99 (52)	–
	Combination	89 (47)	–
	None	3 (2)	–
Treatment: Biologic experienced	DMARD	–	51 (30)
	Biologic	–	25 (15)
	DMARD – biologic	–	94 (55)
DAS28-ESR		4.0 ± 0.5	4.1 ± 0.5
28-TJC		4.3 ± 4.3	4.9 ± 4.2
28-SJC		2.1 ± 2.8	2.7 ± 2.6
PtGA		50.0 ± 21.8	51.0 ± 21.4
ESR		21.8 ± 16.3	20.0 ± 16.1
HAQ-DI		1.2 ± 0.8	1.5 ± 0.7
EQ-5D-3L index		0.5 ± 0.3	0.5 ± 0.3
EQ-5D-3L pain	None	12 (7)	8 (4)
	Moderate	135 (79)	153 (82)
	Severe	23 (14)	26 (14)
EQ-5D-3L anxiety/depression	None	92 (55)	91 (49)
	Moderate	64 (38)	87 (47)
	Severe	12 (7)	8 (4)

Results are presented as n (%) or mean ± standard deviation. The following data are missing in the biologic-naïve population: ethnicity in 9 patients; disease duration in 7 patients; RF in 43 patients; anti-CCP in 80 patients; DMARD treatment in 16 patients; TJC/SJC/PtGA in 1 patient; ESR in 6 patients; EQ-5D-3L index scores in 35 patients. The following data are missing in the biologic-experienced population: sex in 2 patients; ethnicity in 37 patients; RF in 25 patients; anti-CCP in 78 patients; DMARD treatment in 18 patients; PtGA in 1 patient; ESR in 12 patients; EQ-5D-3L index scores in 1 patient

*Abbreviations: CCP* cyclic citrullinated peptide antibody, *DAS28-ESR* Disease Activity Score for 28-joint count based on erythrocyte sedimentation rate, *DMARD* disease-modifying anti-rheumatic drug, *EQ-5D-3L* 3-level EuroQol 5-dimensions questionnaire, *ESR* erythrocyte sedimentation rate, *HAQ-DI* Health Assessment Questionnaire-Disability Index, *PtGA* patient global assessment, *RA* rheumatoid arthritis, *RF* rheumatoid factor, *SJC* swollen joint count, *TJC* tender joint count

### Changes in HAQ-DI scores

Baseline and 12-month mean HAQ-DI scores were similar in the two patient groups. In biologic-naïve patients, baseline and 12-month mean HAQ-DI scores were 1.21 ± 0.79 (standard deviation [SD]) and 1.21 ± 0.80, respectively. Many patients showed substantial changes in HAQ-DI scores over the follow-up period (Supplementary Fig. 1). A worsening or improvement in HAQ-DI scores that exceeded the minimum clinically important difference (MCID) of 0.22 units [14] was observed in 60 (29%) and 58 (28%) patients, respectively. In biologic-experienced patients, baseline and 12-month mean HAQ-DI scores were 1.46 ± 0.74 and 1.47 ± 0.76, respectively. A worsening or improvement in HAQ-DI scores that exceeded the MCID of 0.22 units was observed in 56 (30%) and 50 (27%) patients, respectively.

### Changes in EQ-5D-3L index scores

As with HAQ-DI scores, baseline and 12-month mean EQ-5D-3L index scores were similar in both patient groups. In biologic-naïve patients, baseline and 12-month mean EQ-5D-3L index scores were 0.52 ± 0.30 (SD) and 0.53 ± 0.30, respectively; mean change in EQ-5D-3L index scores was 0.02 ± 0.31, reflecting an improvement in QoL. In biologic-experienced patients, baseline and 12-month mean EQ-5D-3L index scores were 0.50 ± 0.26 and 0.49 ± 0.31, respectively; mean change in EQ-5D-3L index scores was minimal (− 0.005 ± 0.31). Despite small changes in mean scores, histograms demonstrated that many patients showed substantial changes in EQ-5D-3L index scores over the follow-up period (Supplementary Fig. 1).

### Predictors of endpoint HAQ-DI scores

In univariate analysis in the biologic-naïve group, baseline age, male sex, disease duration, RF, DAS28-ESR, SJC, PtGA, HAQ-DI, EQ-5D-3L index scores, EQ-5D-3L pain scores, and EQ-5D-3L anxiety/depression scores all showed significant associations (*P* < 0.05) with 12-month HAQ-DI scores (Table 2). In the biologic-experienced group, age, corticosteroid use, SJC, PtGA, HAQ-DI, EQ-5D-3L index scores, EQ-5D-3L pain scores, and EQ-5D-3L anxiety/depression scores all showed significant associations (*P* < 0.05) with 12-month HAQ-DI scores (Table 3).

**Table 2:** Association of baseline variables with HAQ-DI and EQ-5D-3L index scores in biologic DMARD-naïve patients

Baseline variable	12-month HAQ-DI score				12-month change in HAQ-DI score				12-month EQ-5D-3L score				12-month change in EQ-5D-3L score			
	Univariate		Multivariate		Univariate		Multivariate		Univariate		Multivariate		Univariate		Multivariate	
	β	*P*	β	*P*	β	*P*	β	*P*	β	*P*	β	*P*	β	*P*	β	*P*
Age	0.01	**0.017**	0.00	0.284	0.00	**0.084**	0.00	**0.035**	0.00	0.595	–	–	0.00	0.849	–	–
Male sex	−0.36	**0.010**	−0.19	0.134	−0.01	0.948	–	–	0.24	0.182	–	–	0.30	0.149	–	–
White	Ref	–	–	–	Ref	–	–	–	Ref	–	Ref	–	Ref	–	Ref	–
Black	0.25	0.105	–	–	−0.06	0.547	–	–	−0.52	**0.013**	−0.44	**0.017**	−0.12	0.086	−0.16	**0.010**
Asian	−0.39	0.113	–	–	−0.15	0.354	–	–	0.34	0.246	−0.15	0.571	0.07	0.495	0.06	0.506
Mixed	−0.17	0.675	–	–	−0.04	0.888	–	–	0.47	0.329	0.21	0.610	0.24	0.139	0.16	0.268
Other	0.16	0.571	–	–	0.17	0.357	–	–	−0.61	0.100	−0.52	0.095	−0.08	0.523	−0.13	0.207
Disease duration	0.01	**0.037**	0.01	0.208	0.01	0.197	–	–	0.00	0.941	–	–	0.00	0.854	–	–
RF positive	−0.45	**< 0.001**	−0.19	0.051	−0.06	0.486	–	–	0.07	0.708	–	–	−0.10	0.102	–	–
Corticosteroids	−0.02	0.906	–	–	0.05	0.685	–	–	0.23	0.365	–	–	0.30	0.710	–	–
DAS28-ESR	0.21	**0.048**	−0.01	0.881	−0.08	0.254	–	–	−0.05	0.708	–	–	0.05	0.282	–	–
Swollen joint count	−0.06	**0.002**	−0.02	0.227	−0.01	0.415	–	–	0.08	**0.006**	0.04	0.184	0.00	0.664	–	–
Tender joint count	0.01	0.637	–	–	0.00	0.926	–	–	0.00	0.853	–	–	0.00	0.576	–	–
PtGA	0.01	**< 0.001**	0.00	0.225	0.00	**0.008**	0.00	0.643	−0.01	**< 0.001**	0.00	0.502	0.00	0.069	0.00	0.197
ESR	0.00	0.626	–	–	0.00	0.971	–	–	0.00	0.678	–	–	0.00	0.662	–	–
DMARD monotherapy	Ref	–	–	–	Ref	–	–	–	Ref	–	Ref	–	Ref	–	–	–
DMARD combination	0.05	0.646	–	–	0.07	0.340	–	–	−0.40	**0.008**	−0.44	**0.002**	−0.08	0.138	–	–
No DMARDs	−0.65	0.171	–	–	−0.10	0.738	–	–	−0.01	0.989	−0.48	0.300	−0.26	0.160	–	–
HAQ-DI	0.79	**< 0.001**	0.67	**< 0.001**	−0.21	**< 0.001**	−0.21	**< 0.001**	−0.65	**< 0.001**	−0.57	**< 0.001**	0.01	0.739	–	–
EQ-5D-3L index	−1.30	**< 0.001**	−0.45	0.119	0.00	0.977	–	–	1.52	**< 0.001**	0.27	0.527	−0.54	**< 0.001**	−0.73	**< 0.001**
EQ-5D-3L pain	0.47	**< 0.001**	−0.17	0.272	−0.09	0.347	–	–	−0.76	**< 0.001**	−0.11	0.623	0.20	**< 0.001**	−0.05	0.497
EQ-5D-3L anxiety/depression	0.34	**< 0.001**	0.11	0.242	0.003	0.621	–	–	−0.56	**< 0.001**	−0.56	0.089	0.10	**0.008**	−0.04	0.353

β- and *P*-values are from linear regression models that included endpoint HAQ-DI score, endpoint EQ-5D-3L index score (rank-transformed), 12-month change in HAQ-DI score or 12-month change in EQ-5D-3L index score as the response variable and each baseline variable as the explanatory variable (univariate model) or all baseline variables with *P*-values < 0.1 from univariate analysis (multivariate model); *P*-values shown in bold are significant (< 0.05)

*Abbreviations: DAS28-ESR* Disease Activity Score for 28-joint count based on erythrocyte sedimentation rate, *DMARD* disease-modifying anti-rheumatic drug, *EQ-5D-3L* 3-level EuroQol 5-Dimensions questionnaire, *ESR* erythrocyte sedimentation rate, *HAQ-DI* Health Assessment Questionnaire-Disability Index, *PtGA* Patient Global Assessment of Disease Activity on a 10 cm visual analogue scale, *Ref* reference group, *RF* rheumatoid factor

**Table 3:** Association of baseline variables with HAQ-DI and EQ-5D-3L index scores in biologic DMARD-experienced patients

Baseline variable	12-month HAQ-DI score				12-month change in HAQ-DI score				12-month EQ-5D-3L score				12-month change in EQ-5D-3L score			
	Univariate		Multivariate		Univariate		Multivariate		Univariate		Multivariate		Univariate		Multivariate	
	β	*P*	β	*P*	β	*P*	β	*P*	β	*P*	β	*P*	β	*P*	β	*P*
Age	0.01	**0.003**	0.00	0.554	0.00	0.698	–	–	0.00	0.510	–	–	0.00	0.510	–	–
Male sex	−0.06	0.679	–	–	0.07	0.468	–	–	−0.30	0.119	–	–	0.01	0.886	–	–
White	Ref	–	–	–	Ref	–	–	–	Ref	–	Ref	–	Ref	–	Ref	–
Black	−0.23	0.173	–	–	−0.08	0.527	–	–	0.12	0.574	–	–	0.03	0.620	–	–
Asian	−0.39	0.218	–	–	−0.10	0.658	–	–	0.17	0.672	–	–	−0.08	0.542	–	–
Mixed	−0.43	0.328	–	–	−0.07	0.813	–	–	0.66	0.341	–	–	0.05	0.815	–	–
Other	−0.05	0.888	–	–	−0.26	0.291	–	–	0.51	0.255	–	–	0.23	0.106	–	–
Disease duration	0.00	0.667	–	–	0.00	0.845	–	–	0.01	0.395	–	–	0.00	0.862	–	–
RF positive	−0.14	0.342	–	–	−0.12	0.249	–	–	0.12	0.535	–	–	0.02	0.730	–	–
Corticosteroids	0.31	**0.026**	0.09	0.369	0.00	0.968	–	–	−0.12	0.490	–	–	0.03	0.572	–	–
DAS28-ESR	0.00	0.986	–	–	−0.14	0.057	0.01	0.891	0.00	0.990	–	–	0.09	0.053	0.05	0.269
Swollen joint count	−0.04	**0.049**	−0.01	0.391	0.00	0.991	–	–	0.04	0.138	–	–	0.01	0.510	–	–
Tender joint count	−0.02	0.139	–	–	−0.02	**0.001**	−0.03	**0.006**	0.01	0.572	–	–	0.01	0.161	–	–
PtGA	0.01	**0.014**	0.00	0.401	0.00	0.060	0.00	0.421	−0.01	**0.028**	0.00	0.850	0.00	**0.047**	0.00	0.293
ESR	0.00	0.628	–	–	0.00	0.754	–	–	0.00	0.222	–	–	0.00	0.774	–	–
DMARD	Ref	–	–	–	Ref	–	–	–	Ref	–	Ref	–	Ref	–	Ref	–
Biologic	0.11	0.561	–	–	0.08	0.530	–	–	0.08	0.899	–	–	−0.04	0.604	–	–
DMARD biologic	0.02	0.902	–	–	−0.01	0.882	–	–	−0.02	0.899	–	–	−0.04	0.472	–	–
HAQ-DI	0.79	**< 0.001**	0.76	**< 0.001**	−0.21	**< 0.001**	−0.21	**< 0.001**	− 0.49	**< 0.001**	−0.29	**0.004**	0.03	0.283	–	–
EQ-5D-3L index	−1.08	**< 0.001**	−0.02	0.949	0.28	0.053	−0.10	0.546	1.61	**< 0.001**	1.18	**0.007**	−0.50	**< 0.001**	−0.40	**0.003**
EQ-5D-3L pain	0.33	**0.012**	0.01	0.935	−0.11	0.211	–	–	−0.53	**0.002**	0.23	0.325	0.29	**< 0.001**	0.13	0.090
EQ-5D-3L anxiety/depression	0.23	**0.019**	0.05	0.544	−0.01	0.875	–	–	−0.54	**< 0.001**	−0.25	0.058	0.09	**0.026**	−0.02	0.674

β- and *P*-values are from linear regression models that included endpoint HAQ-DI score, endpoint EQ-5D-3L index score (rank-transformed), 12-month change in HAQ-DI score or 12-month change in EQ-5D-3L index score as the response variable and each baseline variable as the explanatory variable (univariate model) or all baseline variables with *P*-values < 0.1 from univariate analysis (multivariate model); *P*-values shown in bold are significant (< 0.05)

*Abbreviations: DAS28-ESR* Disease Activity Score for 28-joint count based on erythrocyte sedimentation rate, *DMARD* disease-modifying anti-rheumatic drug, *EQ-5D-3L* 3-level EuroQol 5-Dimensions questionnaire, *ESR* erythrocyte sedimentation rate, *HAQ-DI* Health Assessment Questionnaire-Disability Index, *PtGA* Patient Global Assessment of Disease Activity on a 10 cm visual analogue scale, *Ref* reference group, *RF* Rheumatoid factor

**Fig. 1:**
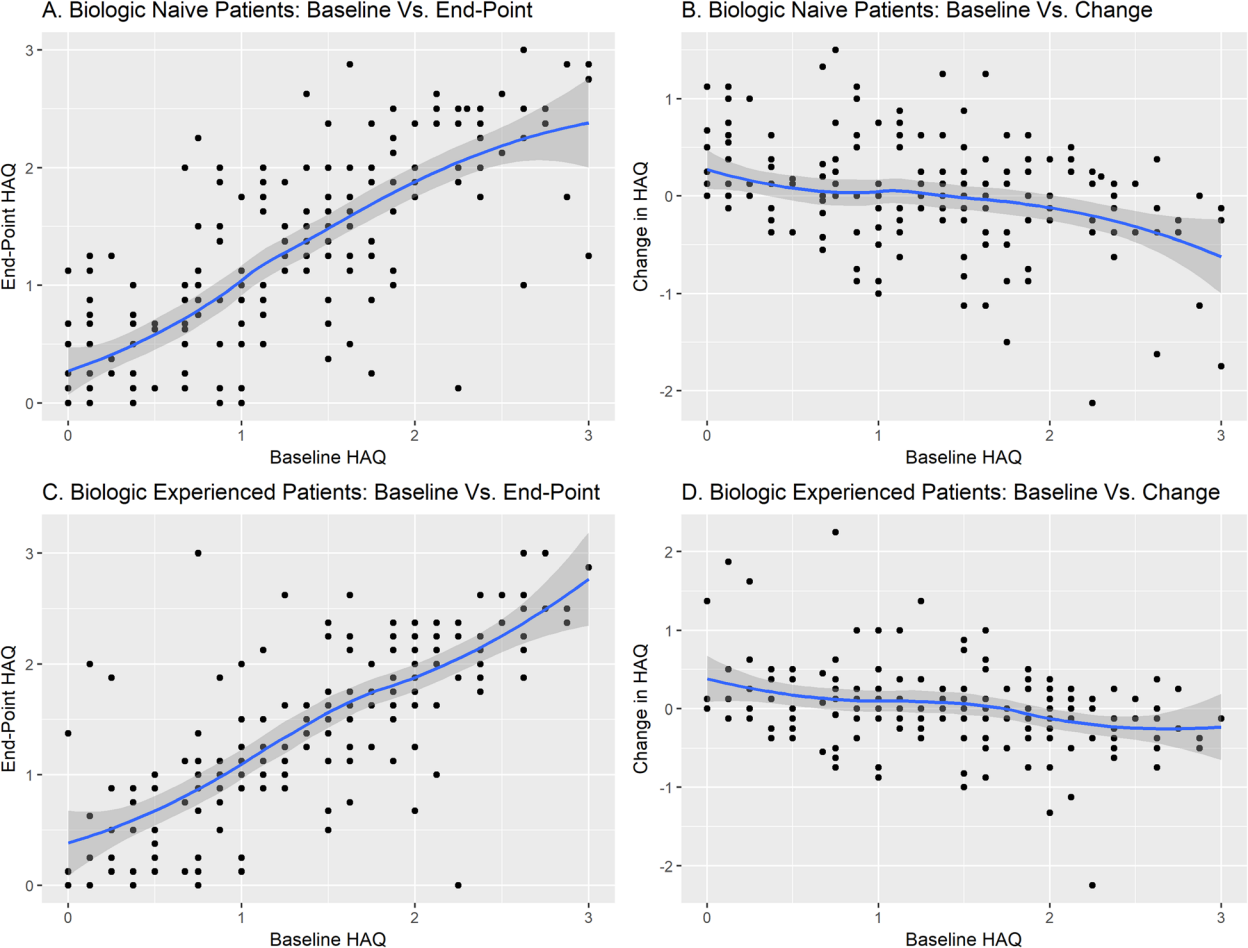
Baseline vs. 12-month HAQ-DI scores in biologic-naïve (**a, b**) and biologic-experienced (**c, d**) patients. Scatterplots show baseline Health Assessment Questionnaire-Disability Index (HAQ-DI) scores vs. 12-month HAQ-DI scores or 12-month change in HAQ-DI scores for biologic-naïve (N = 207) and biologic-experienced (*N* = 188) patients

In multivariate analysis in both patient groups, only baseline HAQ-DI score showed a significant association with 12-month HAQ-DI scores, with higher baseline HAQ-DI scores being associated with higher 12-month HAQ-DI scores, indicating greater disability (Tables 2 and 3, and Fig. 1). In biologic-naïve patients, the β-value of 0.67 (standard error [SE] 0.07) indicated that the 12-month HAQ-DI score was 0.67 units higher per unit increase in baseline HAQ-DI score. In biologic-experienced patients, the β-value of 0.76 (SE 0.06) indicated that the 12-month HAQ-DI score was 0.76 units higher per unit increase in baseline HAQ-DI score.

### Predictors of change in HAQ-DI scores

In univariate analysis in the biologic-naïve group, age, PtGA, and HAQ-DI scores all showed significant associations (*P* < 0.05) with 12-month changes in HAQ-DI scores (Table 2), whereas variables associated with such changes in the biologic-experienced group were DAS28-ESR, TJC, PtGA, and HAQ-DI scores (Table 3).

In multivariate analysis in both patient groups, baseline HAQ-DI score showed a significant association with 12-month changes in HAQ-DI scores, with higher baseline HAQ-DI scores predicting greater 12-month reductions (improved functioning) in HAQ-DI scores (Tables 2 and 3, and Fig. 1). In both biologic-naïve and biologic-experienced patients, the β-values of − 0.21 (SE 0.05) and − 0.21 (SE 0.06), respectively, indicated that the 12-month reduction in HAQ-DI score was 0.21 units greater per unit increase in the baseline HAQ-DI score. Significant associations with 12-month changes in HAQ-DI scores were also observed for age (*P* = 0.035) in biologic-naïve patients and for TJC (*P* = 0.006) in biologic-experienced patients; the small β-values (0.005 and − 0.03, respectively) indicated uncertain clinical relevance for these statistical associations.

### Predictors of endpoint EQ-5D-3L index scores

**Fig. 2:**
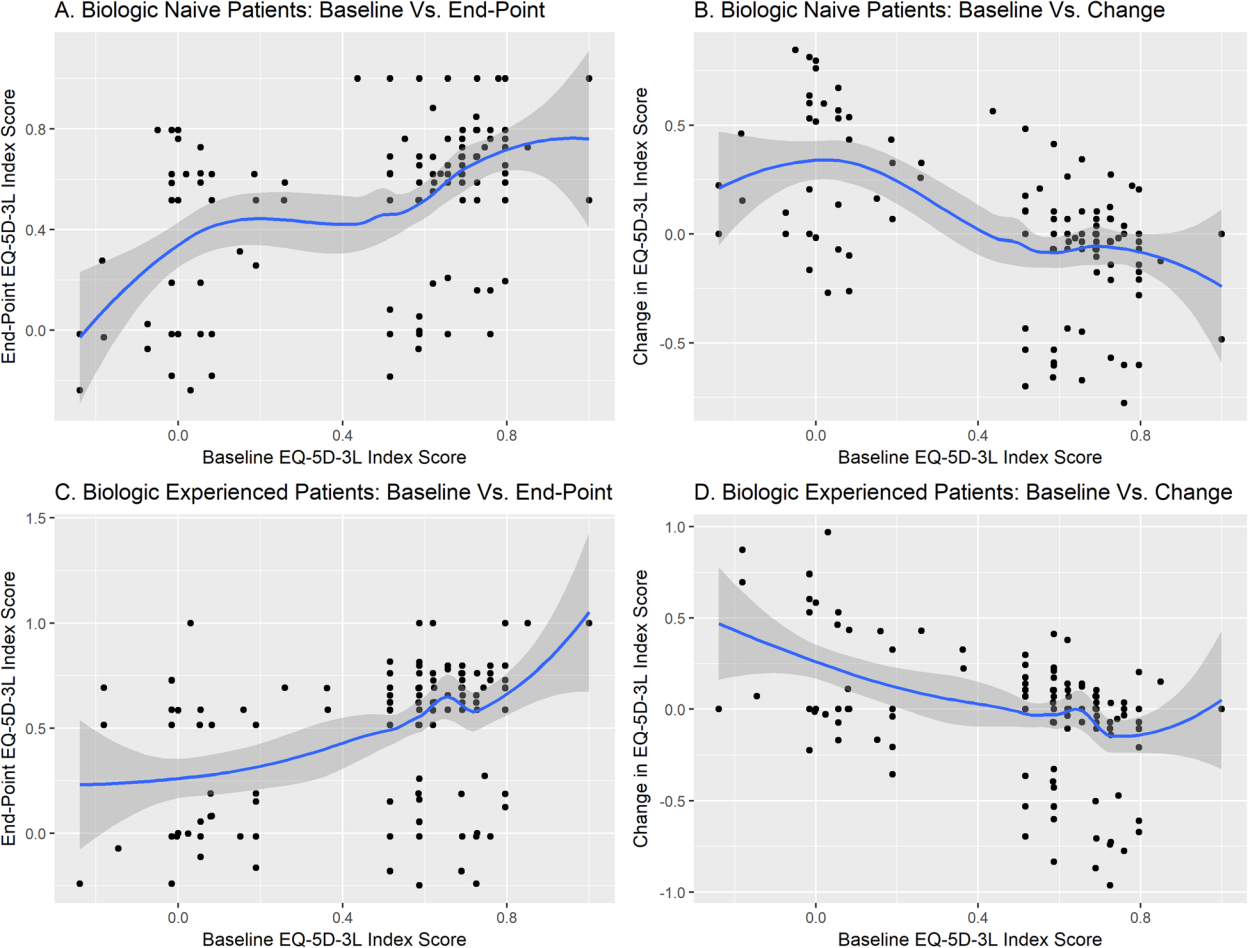
Baseline vs. 12-month EQ-5D-3L index scores in biologic-naïve (**a, b**) and biologic-experienced (**c, d**) patients. Scatterplots show baseline EuroQol-5 dimensions questionnaire, 3-level version (EQ-5D-3L) index scores vs. 12-month EQ-5D-3L index scores or 12-month change in EQ-5D-3L index scores for biologic-naïve (*N* = 207) and biologic-experienced (*N* = 188) patients

In univariate analysis in both patient groups, PtGA, HAQ-DI, EQ-5D-3L index scores, EQ-5D-3L pain scores, and EQ-5D-3L anxiety/depression scores all showed significant associations (*P* < 0.05) with 12-month EQ-5D-3L index scores (Tables 2 and 3; Fig. 2). In the biologic-naïve group, Black ethnicity, the use of combination DMARD therapy, and SJC also showed significant associations.

In multivariate analysis, baseline HAQ-DI scores were significantly associated with 12-month EQ-5D-3L index scores in both biologic-naïve (β = − 0.57; SE 0.10) and biologic-experienced patients (β = − 0.29). It was not possible to clinically interpret the β-values from the regression models because of the rank-transformation of 12-month EQ-5D-3L index scores. Twelve-month EQ-5D-3L index scores had significant associations with Black ethnicity and combination DMARD therapy in biologic-naïve patients and with baseline EQ-5D-3L index scores in biologic-experienced patients.

### Predictors of change in EQ-5D-3L index scores

In univariate analysis in both patient groups, EQ-5D-3L index scores, EQ-5D-3L pain scores, and EQ-5D-3L anxiety/depression scores all showed significant associations (*P* < 0.05) with 12-month changes in EQ-5D-3L index scores (Tables 2 and 3; Fig. 2). PtGA also showed a significant association in the biologic-experienced group.

In multivariate analysis, baseline EQ-5D-3L scores were significantly associated with 12-month changes in EQ-5D-3L index scores in both patient groups. In biologic-naïve patients, the β-value of − 0.73 (SE 0.14) indicated that the 12-month decrease in EQ-5D-3L index scores was 0.73 units more per unit increase in the baseline EQ-5D-3L index score. In biologic-experienced patients, the β-value of − 0.40 (SE 0.13) indicated that the 12-month decrease in EQ-5D-3L index scores was 0.40 units more per unit increase in the baseline EQ-5D-3L index score. In biologic-naïve patients, Black ethnicity also showed a significant association with 12-month changes in EQ-5D-3L index scores (β = − 0.16; *P* = 0.010).

## Discussion

Our retrospective analysis of patients with persistent MDAS in a real-world, T2T setting has three key findings. First, it shows HAQ-DI is a key predictor of patients’ future functional status and HRQoL, with baseline HAQ-DI scores significantly associated with 12-month HAQ-DI and EQ-5D-3L index scores in both biologic-naïve and biologic-experienced patients. Second, it supports existing research indicating that MDAS is not a benign disease activity state; many patients evaluated in this study had substantial ongoing impaired physical function and HRQoL. Third, it highlights the ongoing unmet need to address pain in patients with RA; over 90% of patients reported moderate or severe pain when assessed using the EQ-5D-3L. Overall, these findings support the T2T strategy of aiming for remission/LDAS [2, 3] and the current UK National Institute for Health and Care Excellence (NICE) guidelines advocating use of the HAQ-DI in routine care [15]. Achieving MDAS alone appears insufficient to optimise pain relief, physical function, and HRQoL.

Over 12 months, mean HAQ-DI scores were both relatively high and relatively static in patients with MDAS. This finding is consistent with data from the Yorkshire Early Arthritis Register, which showed that persistent MDAS was associated with persistently high HAQ-DI scores in early RA [6]. However, a minority of individuals did demonstrate large changes in their HAQ-DI scores, reflecting evidence that individual patients have different HAQ-DI trajectories, both in early disease [16] and in persistent MDAS after biologic therapy [17]. An associated finding in our study was that initial HAQ-DI scores strongly predicted final HAQ-DI scores, patients with worse baseline function tending to have worse function at study endpoint. This finding reflects the considerable evidence that baseline HAQ-DI scores are important predictors of subsequent outcomes, including future disability. Research on HAQ-DI as a predictor of outcomes has been mainly performed in early RA cohorts [18–21], though there is evidence that baseline HAQ-DI scores are also predictors in established RA [22]. Our further finding that higher baseline HAQ-DI scores were significantly associated with greater 12-month reductions in HAQ-DI scores, which appears counterintuitive, is most likely explained by ‘regression to the mean’. This well-established and ubiquitous statistical phenomenon occurs when unusually large or small measurements tend to be followed by measurements that are closer to the mean [23], and is seen throughout studies using repeated measures, including birthweight of subsequent childbirths [24], blood pressure [25], bone mineral density scores [26] and cholesterol levels [27].

We found that most MDAS patients (93 and 96% of biologic DMARD-naïve and biologic DMARD-experienced patients, respectively) had moderate to severe pain on the EQ-5D-3L pain scale at baseline. This supports existing patient survey data highlighting that pain is a significant unmet need for patients with RA, with many patients reporting pain to be their preferred area for improvement in the management of their disease [28–30]. Pain in people with RA is a multidimensional experience driven by a broad range of factors, including disease activity, pain pathway sensitisation, joint damage, and the health beliefs of patients [31]. The cornerstone of pain management lies in its assessment, with Chua et al. [32] recommending the collection of pain scores at all clinic assessments. Our data support their suggestion, and further underline the importance of improving how pain is managed in people with RA.

Our study has several strengths. These include the real-life T2T clinical setting, the focus on a previously poorly studied patient population in persistent MDAS, and the comprehensive prospective capture of clinical data over an extended period. It also has several limitations. First, the use of data from a single centre resulted in a relatively limited sample size. In addition, these data may not be representative of all rheumatology centres in the UK. Small sample sizes are an inevitable consequence of single-centre studies, since the numbers of patients in specific disease activity groups are likely to be restricted. However, single centres can also achieve a consistent management approach, which may be diluted in multicentre observational studies. Second, as these data were captured in a real-life setting when patients routinely attended the rheumatology clinic rather than scheduled study visits, there was also some variability in the times at which outcome variables were measured. Such variability is unavoidable in real-life settings. Third, pain was assessed using the EQ-5D-3L pain scale, which only evaluates one facet of a multidimensional problem using a limited number of pain categories.

## Conclusion

The HAQ-DI is an important predictor of future function and HRQoL. Our study underlines the need to supplement disease activity assessments with HAQ-DI measurements (in-line with existing NICE guidelines), to provide important insight into which patients are likely to demonstrate poor future function and reduced HRQoL. It also provides further evidence that persistent MDAS is not a ‘benign’ disease activity state, with many patients in our study experiencing substantial physical disability and poor HRQoL alongside high pain levels.

## Supplementary information

**Additional file 1 Supplementary Fig. 1.** Changes in study variables in biologic-naïve (A, B, C) and biologic-experienced (C, D, E) patients. Legend: Histograms show 12-month changes in DAS28-ESR, HAQ-DI, and EQ-5D-3L index scores in biologic-naïve (*N* = 207) and biologic-experienced (*N* = 188) patients.

## Data Availability

The datasets generated and/or analysed during the current study are not publicly available as they represent routinely collected NHS data from a single centre, leading to the potential for patient confidentiality to be compromised.

## Abbreviations

DAS28: Disease Activity Score for 28-joint count
DAS28-ESR: Disease Activity Score for 28-joint count based on erythrocyte sedimentation rate
DMARD: Disease-modifying anti-rheumatic drug
EQ-5D-3L: 3-level version of the EuroQol 5-dimensions questionnaire
ESR: Erythrocyte sedimentation rate
HAQ-DI: Health Assessment Questionnaire-Disability Index
HDAS: High disease activity state
HRQoL: Health-related quality of life
LDAS: Low disease activity state
MCID: Minimum clinically important difference
MDAS: Moderate disease activity state
NICE: National Institute for Health and Care Excellence
PtGA: Patient Global Assessment of Disease Activity
QoL: Quality of life
RA: Rheumatoid arthritis
RF: Rheumatoid factor
RAPID3: Routine Assessment of Patient Index Data 3
SD: Standard deviation
SE: Standard error
SJC: Swollen joint count
TJC: Tender joint count
T2T: Treat-to-target
