# Supplementary figures and images for: Factors associated with disability in patients with rheumatoid arthritis with persistent moderate disease activity: a retrospective cohort study
Source: BMC Rheumatol. 2020 Oct 21;4:63. doi: 10.1186/s41927-020-00161-4 (PMC7576705; doi:10.1186/s41927-020-00161-4)

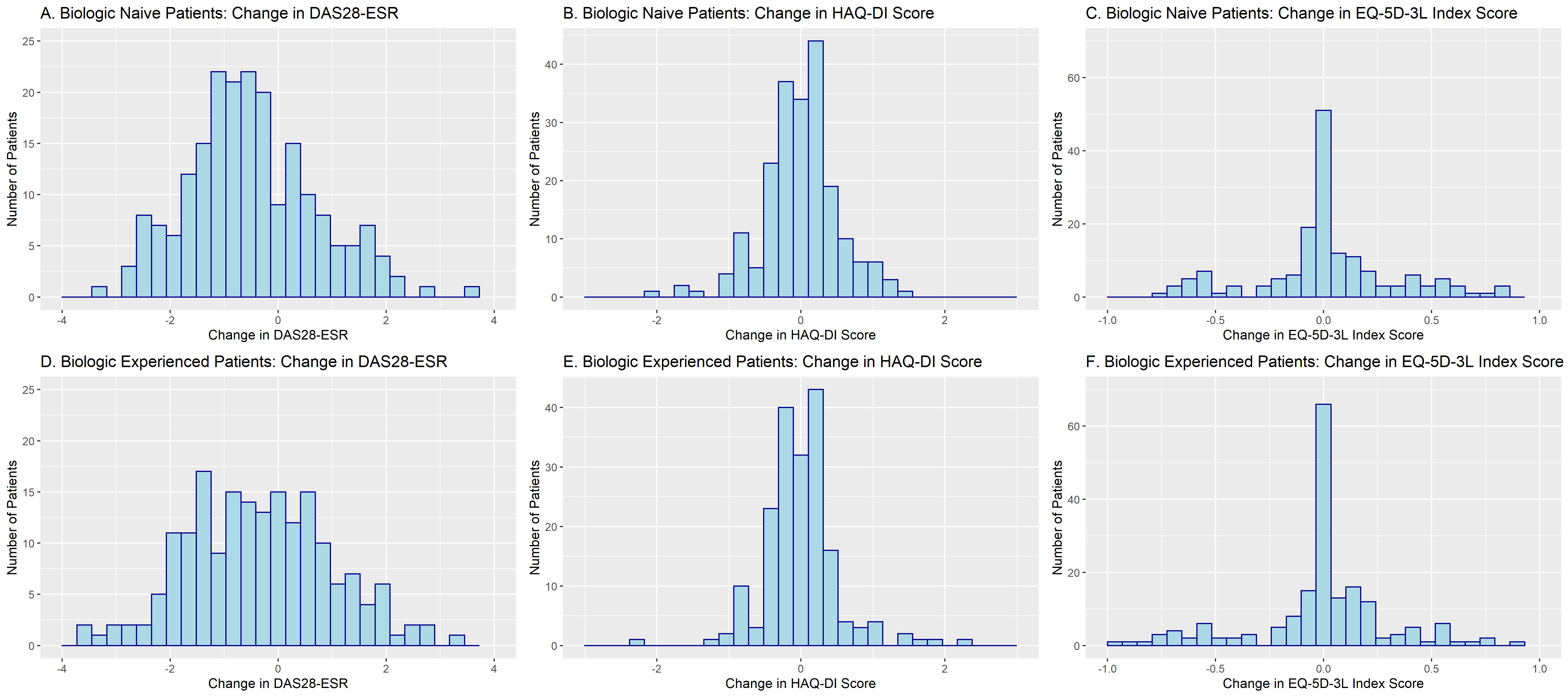

Supplement: Supplementary file 1 — Additional file 1 Supplementary Fig. 1. Changes in study variables in biologic-naïve (A, B, C) and biologic-experienced (C, D, E) patients. Legend: Histograms show 12-month changes in DAS28-ESR, HAQ-DI, and EQ-5D-3L index scores in biologic-naïve (N = 207) and biologic-experienced (N = 188) patients. [file 41927_2020_161_MOESM1_ESM.tiff]
